# Concurrent reactivation of varicella zoster virus and herpes simplex virus in an immunocompetent elderly male^☆^^☆☆^

**DOI:** 10.1016/j.abd.2019.01.003

**Published:** 2019-10-26

**Authors:** Miguel Costa-Silva, Joana Sobrinho-Simões, Filomena Azevedo, Carmen Lisboa

**Affiliations:** a Department of Dermatology and Venereology, Centro Hospitalar Universitário de São João, EPE, Porto, Portugal; b Clinical Pathology Department, Centro Hospitalar Universitário de São João, EPE, Porto, Portugal; c Microbiology Department, Faculty of Medicine, University of Porto, Porto, Portugal

Dear Editor,

Cutaneous infections by herpes simplex virus (HSV) and varicella zoster virus (VZV), both of which belong to the alpha subfamily of herpes viruses, are relatively common.^1^ After the primary infection, both HSV and VZV remain latent in the nerve tissue for a lifetime and may reactivate.^2^ It has been shown that HSV and VZV may remain latent in the same nerve ganglion.^2^ However, simultaneous reactivation of both HSV and VZV is rare. Giehl et al. found that 20 (1.2%) out of 1718 patients with clinical herpes viruses infection were infected with both HSV and VZV.^1^

A 78-year-old man presented to our dermatology clinic with multiple painful vesicular lesions located on the left forearm and left hand (C6–8 dermatomes) and left lumbar region (T12 dermatome), for four days (Figure 1, Figure 2). Prior to our observation, the patient had been treated with topical betamethasone valerate for three days. The patient's medical history was unremarkable besides childhood varicella. Laboratory investigations revealed an elevated level of C-reactive protein of 1.2 mg/dL, suggesting a mild inflammatory reaction; other blood tests, including immunoglobulin levels, hepatitis B virus, hepatitis C virus, human immunodeficiency virus, and syphilis serology were either normal or negative. HSV-1 and VZV-specific IgG were positive, while IgM HSV and VZV were negative. Real-time polymerase chain reaction (PCR) test detected VZV and HSV-1 DNA in the lesions of the left forearm and left hand, and VZV DNA in the lumbar lesions. A seven-day course of valacyclovir 1000 mg 8/8 h PO resulted in complete resolution.

**Figure 1:**
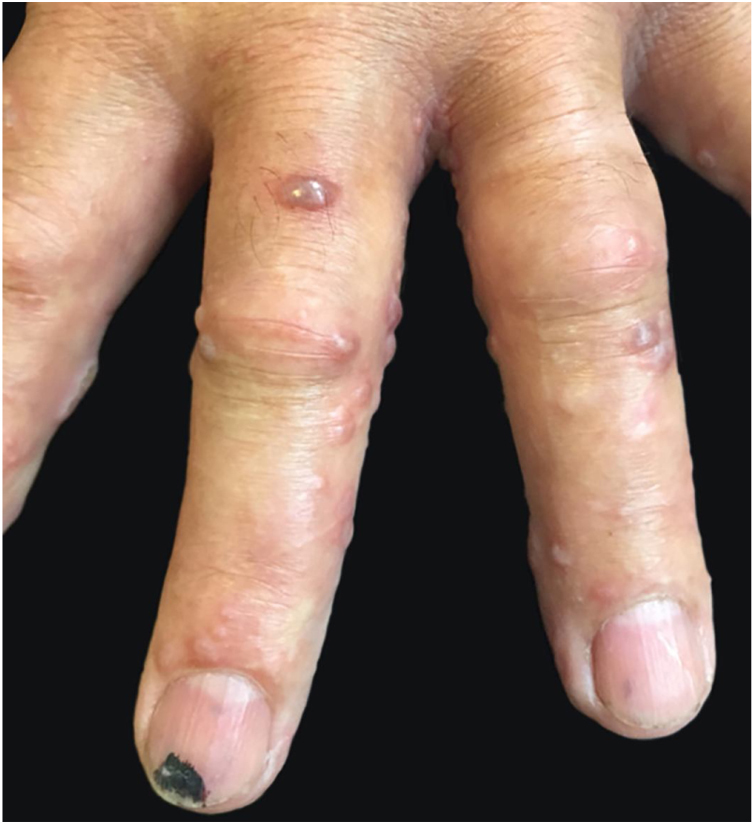
Multiple vesicular lesions affecting the left forearm and left hand.

**Figure 2:**
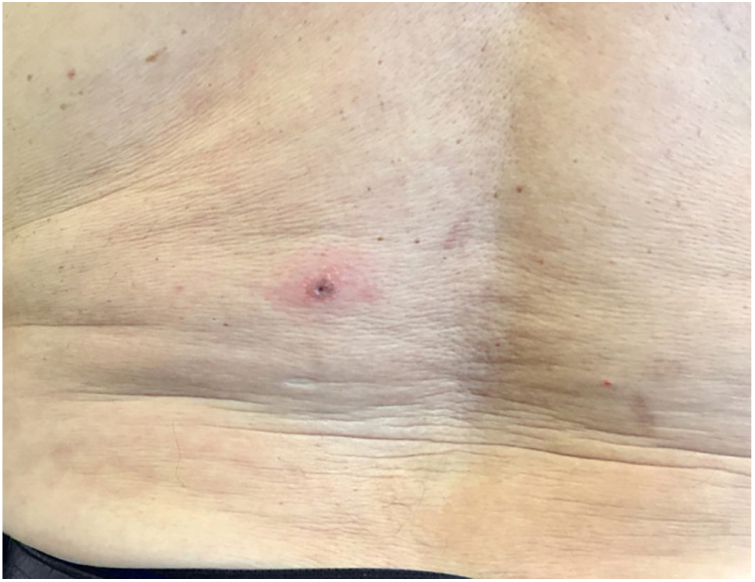
Multiple vesicular lesions affecting the left lumbar region.

HSV and VZV are DNA viruses that share some biologic attributes but, at the same time, differ significantly; such differences may explain why concurrent reactivation is rare.^1^, ^3^ In immunocompetent patients, HSV is reactivated several times during life, but VZV reactivation generally occurs only once.^3^, ^4^ The likelihood of VZV reactivation increases with age, while HSV recurrences decrease with advancing age, probably due to maturation of the immune response.^1^, ^3^ HSV and VZV also differ in their relative capacity to reactivate in response to stimuli that perturb neuronal function.^3^, ^4^ HSV appears to be reactivated by predisposing factors such as UV light exposure, trauma, fever, and stress.^3^, ^4^ Although reports describe induction of zoster with trauma and radiation, VZV does not consistently reactivate in response to recognizable stimuli.^3^, ^4^

Concurrent reactivation of VZV and HSV is possible in both immunocompetent and immunosuppressed patients, although it is more common in the latter group.^1^ It may occur at different sites of the body or at the same location, and it appears to be more common in those aged ≥50 years of age.^1^ Herpes simplex may precede, present simultaneously with, or follow the zoster skin lesions. Medical treatment should be initiated as in the zoster protocol.^2^

We report a case of concurrent reactivation of VZV and HSV in an immunocompetent elderly male with no clinical history of herpes simplex but with serologic evidence of past infection. The combination of high sensitivity and specificity, low contamination risk, and speed has made real-time PCR technology an excellent testing method for diagnosing many infectious diseases. The closed system for amplification and detection used with real-time PCR virtually eliminates amplicon contamination.^5^ Furthermore, the target gene for HSV and VZV detection are different, eliminating the possibility of cross-over reaction.^5^ In this clinical case, the results by real time PCR were strongly positive, and after repetition, they confirmed the result. The PCR test allowed detection and identification of the viral DNA; this knowledge may contribute to the understanding of the pathophysiology of these latent infections.

Simultaneous infection with VZV and HSV was suspected due to the atypical clinical presentation. Dermatologists must be aware of this possibility to assure the correct diagnosis is obtained and that the appropriate treatments are performed.

## Financial support

None declared.

## Author's contributions

Miguel Costa-Silva: Approval of the final version of the manuscript; conception and planning of the study; elaboration and writing of the manuscript; obtaining, analyzing and interpreting the data; effective participation in research orientation; intellectual participation in propaedeutic and/or therapeutic conduct of the cases studied; critical review of the literature; critical review of the manuscript.

Joana Sobrinho-Simões: Approval of the final version of the manuscript; conception and planning of the study; effective participation in research orientation; intellectual participation in propaedeutic and/or therapeutic conduct of the cases studied; critical review of the manuscript.

Filomena Azevedo: Approval of the final version of the manuscript; conception and planning of the study; intellectual participation in propaedeutic and/or therapeutic conduct of the cases studied; critical review of the manuscript.

Carmen Lisboa: Approval of the final version of the manuscript; conception and planning of the study; effective participation in research orientation; intellectual participation in propaedeutic and/or therapeutic conduct of the cases studied; critical review of the literature; critical review of the manuscript.

## Conflicts of interest

None declared.
